# Analysis of Risk Factors for Amputation in 822 Cases with Acute Arterial Emboli

**DOI:** 10.1100/2012/673483

**Published:** 2012-04-19

**Authors:** Ozgur Dag, Mehmet Ali Kaygın, Bilgehan Erkut

**Affiliations:** Erzurum Regional Training and Research Hospital, Cardiovascular Surgery Department, 25080 Erzurum, Turkey

## Abstract

*Background*. We retrospectively examined the records of 822 patients who underwent a total of 901 operations for acute peripheral arterial occlusion of the upper or lower extremities between 1999 and 2009. We analyzed the effects of atherosclerotic structure, the time of admission to hospital, and re-embolectomies on amputation in the early postoperative period. *Methods*. There were 466 (56.7%) men and 356 (43.3%) women. The time of admission to hospital was in the range of 58 hours. There were lower extremity emboli in 683 (83%). Bypass procedures were done in 27 (3.3%) patients. Fasciotomy, patchplasty, and endarterectomy were made in 19 (2.3%), 9 (1.1%), and 7 (0.8%) patients, respectively. *Results*. Early revision (re-embolectomy) was performed in 77 (9.3%) patients. Amputation was performed in 112 (13.6%) patients. Delay after six hours from the onset of complaints and re-embolectomies increased the risk of amputation and rates. *Conclusion*. If the embolectomy, which is a rapid and easy technique for treatment of acute arterial emboli, is performed by experienced surgeons without delay, the complications associated with the emboli may be prevented. Otherwise, delayed operation and repeated re-embolizations in acute arterial play important roles in morbidity.

## 1. Introduction

Acute arterial emboli are a vascular pathology that can be diagnosed in a short time, and the morbidity and mortality of which decreases through urgent surgery. For emboli to occur, It is necessary to be occurred emboli that an abnormal material (thromboemboli, air, fat, tumor, etc.) can occlude the flow way suddenly. Those materials are mostly caused from heart (through left atrium clot developing due to stasis in non-contractile atrium) [1]. They may also develop due to arterial aneurysms (throwing away the clot in the aneurysm to the peripheral). They are localized in the bifurcation regions where the lumen of the narrows. It happens to extremities (70–80%), brain (20%), and visceral arteries (5–10%) [2]. Fogarty balloon catheter embolectomy and heparin treatment are the major methods in the treatment besides surgical and medical treatment due to underlying etiology in mostly all cases [3, 4]. Balloon catheter embolectomy has lessened the rates of morbidity and mortality in an important degree.

In this study, we investigated the effects of pathology related to arteries like early atherosclerosis for the extremity with acute emboli, the time between the onset of complaints and admission to hospital, and re-embolectomy applications on extremity amputation.

## 2. Materials and Methods

**Figure 1:**
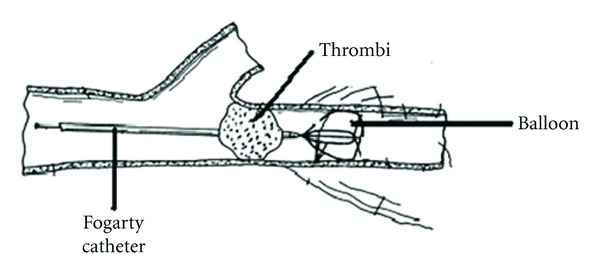
The embolectomy technique with Fogarty catheter.

Early postoperative results of totally 901 surgical operations of 822 patients, who applied to our clinics between May 1999 and January 2009, who were diagnosed as acute arterial emboli, were evaluated. The age range of the patients was between 28 and 96 (mean age was 58), 466 of them were male (56.7%), and 356 were female (43.3%). The period between the onset of complaints and admission to hospital was 2,371 hours (mean duration was 58 hours). While the number of those admitted to hospital earlier than 6 hours was 282 (34.3%), those who admitted later than 6 hours were 540 (65.7%). Though there was lower extremity emboli in 683 (83%) cases, the upper extremity emboli existed only in 99 (12%) cases. In addition, there was diabetes mellitus (DM) in 111 cases (13.5%), chronic obstructive pulmonary disease (COPD) in 55 cases (6.6%) congestive cardiac failure (CCF) in 42 cases (5.2%), and various malignities in 18 cases (2.2%). In all the cases, sudden onset of pain, paleness, chilliness, and pulselessness existed, and in some late cases sensitivity disorders, extremity fatigue, and rarely paralysis were seen. Vascular graft occlusions were emitted from the study. Diagnose was made through physical examination, Doppler ultrasonography, and rarely through classical angiography. Femoral and brachial embolectomy were applied mostly to patients; however, it was necessary to apply from end arteries such as tibialis posterior and radial artery in some cases. Surgery was mostly carried out under local anesthesia. Nevertheless, in some patients whom were decided to undergo revascularization with preoperative graft and due to complications, general anesthesia was needed. Arteriotomy was applied after heparinization and Fogarty catheter was used for embolectomy [5] (Figure 1). Intravenous heparin was administered to all patients postoperatively and if necessary, oral anticoagulant treatment was applied for maintenance.

## 3. Statistical Analysis

“*Q*-square” and “Fisher definite *Q*-square” tests were used in the statistical analysis of qualitative data. The ages of the patients to whom amputation was applied were compared through “Student's *t-*test.” The effective factors on amputation are studied by “logistic regression analysis (the rate of accuracy: 90.67%). In this model, amputation application was considered to be dependent variable, age, sex, arrival time atherosclerosis existence, and application of re-embolectomy were considered to be independent variable. Qualitative data were presented in number and percentage, measurement data in arithmetic mean standard deviation, the results of logistic regression analysis in OR and 95% GA. *P* < 0.05 was accepted to be significant statistically.

## 4. Results

**Figure 2:**
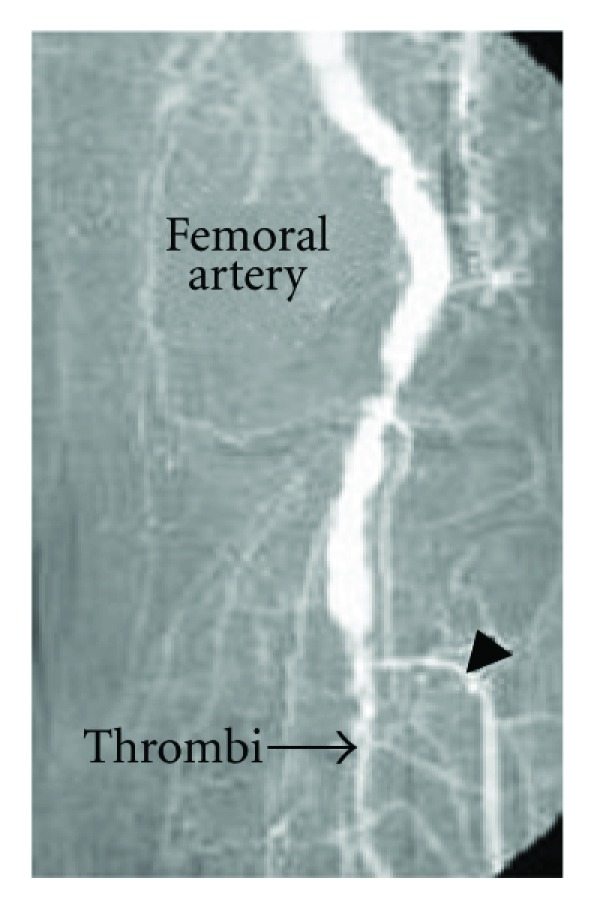
The femoral angiography depicts the right femoral emboli and femoral arterial occlusion.

The underlying reason for emboli in 624 cases (76%) was atrial fibrillation. The sources of emboli are shown in Table 1. Of the cases, 188 (22.8%) underwent right femoral embolectomy (Figure 2), 321 (39%) underwent left femoral embolectomy, 36 (4.3%) right brachial embolectomy, 63 (7.6%) left brachial embolectomy, 28 (3.4%) underwent bilateral femoral embolectomy, 78 (9.4%) right popliteal embolectomy, and 96 (11.6%) underwent left popliteal embolectomy. Four cases (0.4%) were applied right brachial and right femoral embolectomy, and 4 cases (0.4%) were applied left brachial and left femoral embolectomy. Due to the emboli related with popliteal aneurysm in 4 cases (0.4%), femoro-popliteal graft bypass was performed by using saphenous vein. 

Nineteen patients (2.3%) were applied fasciotomy, 27 patients (3.3%) interposition through saphenous vein, 4 patients (0.4%) cross-femoral bypass with synthetic graft, 9 cases (1%) patchplasty, 7 cases (0.8%) endarterectomy, 10 cases (1.2%) arteria tibialis posterior, arteria tibialis anterior additional embolectomy from radial artery, and 2 cases (0.2%) left axillofemoral extra anatomic bypass procedure with synthetic graft.

The mean age of the cases was 58.7 ± 7.5 years. Forty-seven of the female (3.9%) and 65 of the males (14%) underwent amputation (*Q*-square: 0.101, freedom degree: 1, *P* = 0.751). The mean age of the cases undergone amputation (63.4 ± 9.5 years) was statistically higher than those that (50.7 ± 10.7 years) did not undergo (*t* = 4.72, freedom degree: 502, *P* < 0.0005). The patients were followed up during hospitalization, once every 15 days in the postoperative first month, and once a month during the following 6 months.

The effect of the atherosclerotic structure, the duration between the onset of complaints and admission to the hospital and the re-embolectomy on the amputation was statistically evaluated. Amputation was performed in 112 (13.6%) patients. The lower extremity amputation was performed in 89 (79.4%) patients, and upper extremity amputation was performed in 23 (20.6%) cases. Sixteen (2%) patients were lost in the early postoperative period. Fourteen of the cases in whom mortality was seen had CCF, 11 had COPD + CCF, and 13 had CCF + COPD + DM. Atherosclerotic structure was observed in 142 cases (17.2%). Seventy-nine of these (55.6%) needed additional surgical procedure (fasciotomy, endarterectomy, patchplasty, bypass, etc.). Twenty-five (22.3%) of totally 112 amputated cases had atherosclerotic structure. The effect of atherosclerotic structure on amputation was not found to be statistically significant (OR: 1.23, 95% GA: 0.58–2.62, *P* = 0.587). 

The duration between the onset of complaints of the cases and admission to the hospital was 2,371 hours, and the mean duration was 58 hours. Although the number of those admitted to hospital earlier than 6 hours was 282 (34.3%), the number of those admitted later than 6 hours was 540 (65.7%) hours. The number of the cases who admitted to hospital earlier than 6 hours and to whom amputation was performed was only 2 (1.7% of all the amputations) while the number of those admitted hospital later than 6 hours was 101 (90.1% of all the amputations) and the difference between them was statistically significant (OR: 40.3, 95% GA: 5.3–304.9, *P* = 0.0013). The 3 cases necessitated upper extremity amputations were also among those operated later than 6 hours.

Re-embolectomy was performed as a second early period operation in 77 (9.3%) patients. Re-embolectomy was performed to 56 (7.2%) of lower extremity embolectomies, and to 21 (2.5%) of upper extremity embolectomies, and the difference is significant (*Q*-square: 10.12, freedom degree: 1, *P* = 0.001). Forty-nine of the cases (63.6% of all the re-embolectomies) to whom re-embolectomy was performed also underwent amputation, and this value was found to be statistically significant (OR: 42.8, 95% GA: 16.4–111.6, *P* < 0.0005).

The effective factors on amputation were studied through logistic regression analysis. According to the result, the admission time being more than 6 hours (OR: 40.3, 95% GA: 5.3–304, 9, *P* = 0.0013), and re-embolectomy application (OR: 42.8, 95% GA: 16.4–111.6, *P* < 0.0005, increased the risk of amputation Table 2.

## 5. Discussion

Acute arterial emboli are one of the leading operational procedures of vascular surgery. It occurs as a result of blockage of the flow way by an abnormal material such as thrombus, air, fat, or tumor. The cause of many arterial emboli results from the heart [5]. The main reason in rheumatic heart diseases is mitral stenosis and atrial fibrillation [1]. The most frequent cause of emboli in our cases is the blockage of an artery by a heart origin thrombus (through left atrium clot developed due to stasis in noncontractile atrial; 76%), 6P (paresthesia, pain, paleness, paralysis, pulselessness, and prostration) indications are cardinal findings in acute arterial emboli [2]. Sudden pain, paleness, chilliness, and pulselessness were present in all of our cases in addition to loss of sensitivity; extremity fatigue and rarely paralysis were seen in late cases.

It is important to verify the diagnosis of acute arterial emboli. Besides the physical examination, Doppler ultrasonography and angiography may be helpful in diagnosis. Though physical examination was sufficient in our cases, Doppler ultrasonography and angiography were used in rare cases and in some atherosclerotic cases. Although acute emboli can be treated by a simple embolectomy, the development of acute thrombosis under chronic conditions in atherosclerotic cases creates problems from the point of diagnosis and treatment and necessitates additional surgery. We evaluated the degree of ischemia firstly in such cases. If a clinic situation like extremity loss is present in physical examination, the patient was undergone urgent embolectomy from the point of limb salvage by providing preoperative angiography conditions. If ischemia is not in a situation to necessitate extremity loss due to collateral circulation, angiography was performed firstly under affinity, and treated according to the pathology determined. As mentioned above, collateral net causes decrease in amputation rates in patient groups with atherosclerosis. Since atherosclerotic disorder develops in long period, collateral circulation existence gains importance to protect the extremity of the patient. However, bypass procedures are frequently used in these patients during the treatment. In a study presenting the embolectomy process in patients with atherosclerosis postoperative amputation rates were found to be 12.5%, which is better than our results [6]. In another atherosclerotic case group study, worse amputation rate (46%) than our results were found [7]. In the study by Nawaz, amputation rates were found higher in cases with thrombosis [8]. Amputation rate in our cases with atherosclerosis was 22.3% (in 25 out of 112 amputations). Our amputation rates in cases with atherosclerosis do not have a statistical significance (Table 2). In addition, our case studies included more number of cases, a background of cardiac disease and more patients with atherosclerosis than the present study.

Emboli are most frequently seen in femoral and popliteal site in lower extremity and in brachial artery in upper extremity [9]. The sites of embolism in our cases are in accordance with these regions. They are localized in bifurcation region where artery lumen narrows. They happen to extremities with the rate of 70–80%, to brain 20%, and to visceral arteries with the rate of 5–10%. The mean age of cases with arterial embolism changes between 50–65 years [5–10]. In some studies it is indicated as over 60 years, and in some other, there was a case profile in accordance with our age group [11–14]. The mean age of our cases was 58.7 ± 7.5 years.

The future of extremity depends on the degree of ischemia, and the time between the occurrence of embolism and surgery. The most suitable time for interference is the first 6–8 hours. Late embolectomies after acute ischemia may lead to Haimovici-Legrain-Cornier syndrome revascularization syndrome [15]. In such a case, hiperpotasemia, myoglobinuria, and metabolic acidosis are frequently observed [3–5, 9]. Extreme edema occurs during revascularization and a large quantity of free oxygen radicals enters the circulation, and this increases the damage to skeletal muscle. For this reason, it is important to regulate the kidney and heart functions, and liquid and electrolysis impairment in postoperative period. There is significant difference between the development of emboly and arrival at hospital from the point of amputation rates. Englund and Magee reported the amputation rate due to acute arterial emboli as 8.2% [16]. Shifrin et al. indicated that the successful revascularization rate was 70.9% for the patients admitted to hospital in 2–7 days [17]. The number of amputation in those admitted to hospital earlier than 6 hours was 2 (1.7%), and those later than 6 hours was 101 (90.1%), and the difference among them was statistically significant (Table 2). Moreover, 3 cases that necessitated upper extremity amputation were those operated later than 6 hours. As it is understood from the study, late surgeries in acute arterial emboli increase the rate of amputations. It is reported in many studies that amputation rates were in the range of 0–18.9% [13, 16, 18, 19]. The amputation rate in our study was found to be 13.6%. The main reason that increases the amputation rates which are in conformity with literature data is thought to be late admission time.

The occlusion rates leading to re-embolectomy in literature ranges 4–10% [19, 20]. The number of existing re-embolectomies in our cases was 77 (9.3%), and 56 of them belonged to lower extremity. The reasons why the occlusions and thus the re-embolectomies were more than those of literature were the more number of patients with atrial fibrillation, the frequency of repeated attacks, and the more number of patients with atherosclerotic vein structure. If the patient necessitating re-embolectomy had atrial fibrillation, cardiac was again thought to be the source, and thus, there was no need of angiography and re-embolectomy was performed. However, if any prevention or compelling were felt in the arterial wall during the first embolectomy, if there was no predisposition such as atrial fibrillation and KOAH thought to be the source of emboli, and if embolic situation developed again, angiography was performed before re-embolectomy. In addition, there were patients whom were operated or taking preoperative angiography since Fogarty catheter did not go forward during the operation. Forty-nine (63.6%) of the patient to whom re-embolectomy was performed also underwent amputation. This result is significant statistically (Table 2).

## 6. Conclusion

As a result, the rates of amputation are directly related with the time spent between the onset of symptoms and the arterial embolectomy. As mentioned, this ratio may lessen in the patients with atherosclerosis having sufficient collaterals. Amputation was needed in 3 (2.6% of all amputations) cases after upper extremity embolectomy. After the lower extremity embolectomy, 89 cases needed amputation (79.4% of all amputations). This shows us that amputation ratios in ischemia are higher than the emboli related with lower extremity-related embolies, and that prognosis is better for the situations related with upper extremity. Even though amputation develops in upper extremity due to ischemia, it does not lead to pathologies threaten life. Lots of postoperative complications related with thrombectomy can be observed in acute arterial embolies. Hematoma, hemorrhage, and wound-site infection may be the complications to occur. One of the most important of these is repeating embolies, and re-embolectomies to be performed due to these emboli [13, 21].

Finally, due to the advances in modern vascular surgery recently, significant success has been achieved in the diagnosis and treatment of acute arterial embolies. Simple embolectomy performed timely under local anesthesia is the most effective method in the treatment. It was determined in our cases that the duration more than 6 hours between the onset of complaints and operation, and the re-embolectomy increases the risks of amputation when effective factors on amputation are investigated by logistic regression analysis. The diagnosis of the underlying pathology, early diagnosis, and treatment, experience of the team performing the medical and surgical procedures play an important role in the prognosis.

## Figures and Tables

**Table 1:** The sources leading to acute arterial emboli in our study.

Emboli Source	Number of patients	%
Cardiac		
Valve disease (AF)	624	76
Endocarditis	10	1.2
Myxoma	7	0.9
Cardiomyopathy	10	1.2
Cardiac cist	5	0.6
Noncardiac		
Atherosclerosis	142	17.2
Aneurysm sack	4	0.5
Attemptive vascular procedure (surgical)	15	1.8
Phlegmasia cerulea dolens	5	0.6

**Table 2:** Effective factors on amputation (results of logistic regression analysis).

Parameters	Amputation		
	OR	95% GA	*P*
Admission time			
Earlier than 6 hours	1		**0.0013**
Later than 6 hours	40.3	5.3–304.9	
Existence of atherosclerosis			
Absent	1		0.587
Present	1.23	0.58–2.62	
Sex			
Female	1		0.258
Male	0.68	0.35–1.33	
Re-embolectomy			
Not performed	1		**<0.0005**
Performed	42.8	16.4–111.6	
